# Development and head-to-head comparison of machine-learning models to identify patients requiring prostate biopsy

**DOI:** 10.1186/s12894-021-00849-w

**Published:** 2021-05-16

**Authors:** Shuanbao Yu, Jin Tao, Biao Dong, Yafeng Fan, Haopeng Du, Haotian Deng, Jinshan Cui, Guodong Hong, Xuepei Zhang

**Affiliations:** 1 Department of Urology, The First Affiliated Hospital of Zhengzhou University, No. 1 Jianshe East Road, Zhengzhou, 450052 China; 2 Key Laboratory of Precision Diagnosis and Treatment for Chronic Kidney Disease in Henan Province, Zhengzhou, 450052 China

**Keywords:** Prostate cancer, Machine learning, Predictive model, Prostate biopsy

## Abstract

**Background:**

Machine learning has many attractive theoretic properties, specifically, the ability to handle non predefined relations. Additionally, studies have validated the clinical utility of mpMRI for the detection and localization of CSPCa (Gleason score ≥ 3 + 4). In this study, we sought to develop and compare machine-learning models incorporating mpMRI parameters with traditional logistic regression analysis for prediction of PCa (Gleason score ≥ 3 + 3) and CSPCa on initial biopsy.

**Methods:**

A total of 688 patients with no prior prostate cancer diagnosis and tPSA ≤ 50 ng/ml, who underwent mpMRI and prostate biopsy were included between 2016 and 2020. We used four supervised machine-learning algorithms in a hypothesis-free manner to build models to predict PCa and CSPCa. The machine-learning models were compared to the logistic regression analysis using AUC, calibration plot, and decision curve analysis.

**Results:**

The artificial neural network (ANN), support vector machine (SVM), and random forest (RF) yielded similar diagnostic accuracy with logistic regression, while classification and regression tree (CART, AUC = 0.834 and 0.867) had significantly lower diagnostic accuracy than logistic regression (AUC = 0.894 and 0.917) in prediction of PCa and CSPCa (all *P* < 0.05). However, the CART illustrated best calibration for PCa (SSR = 0.027) and CSPCa (SSR = 0.033). The ANN, SVM, RF, and LR for PCa had higher net benefit than CART across the threshold probabilities above 5%, and the five models for CSPCa displayed similar net benefit across the threshold probabilities below 40%. The RF (53% and 57%, respectively) and SVM (52% and 55%, respectively) for PCa and CSPCa spared more unnecessary biopsies than logistic regression (35% and 47%, respectively) at 95% sensitivity for detection of CSPCa.

**Conclusion:**

Machine-learning models (SVM and RF) yielded similar diagnostic accuracy and net benefit, while spared more biopsies at 95% sensitivity for detection of CSPCa, compared with logistic regression. However, no method achieved desired performance. All methods should continue to be explored and used in complementary ways.

## Background

Prostate cancer (PCa) is the most common malignancy of the male reproductive system, with over one million cases and 358 989 deaths in 2018 [1, 2]. Prostate-specific antigen (PSA) testing, introduced in the 1990s, not only increased the incidence of clinically insignificant PCa (CSPCa, defined as Gleason score ≥ 3 + 4), but also led to an increased number of unnecessary biopsies. This is particularly the case in a PSA gray zone, at which 65–70% of men have a negative biopsy result [3]. In our study, the PCa and CSPCa were detected in 23% and 17%, 27% and 18%, 53% and 47%, 68% and 66%, 73% and 68%, and 93% and 93% of the men with serum total PSA (tPSA) in the range of ≤ 10 ng/ml, 10–20 ng/ml, 20–30 ng/ml, 30–40 ng/ml, 40–50 ng/ml, and > 50 ng/ml, respectively. Therefore, the major challenge is to identify CSPCa among cases with serum tPSA ≤ 50 ng/ml at an early stage.

Studies have validated the clinical utility of multiparametric resonance imaging (mpMRI) in the detection and localization of International Society of Urological Pathology grade ≥ 2 cancers [4, 5]. Additionally, predictive models have the potential to improve diagnostic accuracy to influence disease trajectory and reduce healthcare costs [6, 7]. To reduce unnecessary biopsy and overdiagnosis, a dozen of nomograms have been used to help diagnose PCa and/or CSPCa, including PCPT-RC [8], STHLM3 [9], ERSPC-RC [10], and CRCC-PC [11], which are based on standard statistical technique of logistic regression (LR).

Over the past decade, we have entered the era of big data, and major advancements have emerged in the fields of statistics, artificial intelligence technology and urological medicine [12, 13]. Machine learning-assisted models have been proposed as a supplement or alternative for standard statistical techniques, including artificial neural network (ANN), support vector machine (SVM), classification and regression tree (CART), and random forest (RF). Machine learning has many attractive theoretic characteristics, specifically, the ability to deal with non-predefined relations such as nonlinear effects and/or interactions, at the cost of reducing interpretability and explanation, especially for complex nonlinear models [14, 15]. However, model validation helps to discover domain-relevant models with better generalization ability, and further implies better interpretability. These new algorithms incorporating mpMRI parameters may help improve the diagnosis of CSPCa [16, 17], but available data is limited.

In this study, we sought to develop and evaluate of multiple supervised machine-learning models based on age, PSA derivates, prostate volume, and mpMRI parameters to predict PCa and CSPCa. Additionally, we compare our models with conventional LR analysis to evaluate whether there were improvements in the diagnostic ability, using the same variables and population.

## Methods

### Study populations

This retrospective study was approved by the Institutional Ethics Review Board, and a waiver of informed consent was obtained. Between April 2016 and March 2020, prostate biopsy and mpMRI examination was done among 903 consecutive patients without a prior prostate biopsy. The 25 patients diagnosed with other types of tumors, 94 patients with incomplete data, and 96 patients with tPSA > 50 ng/ml were excluded leaving 688 cases available for analysis.

### Data collection

The clinical variables including the age at prostate biopsy, serum tPSA and free PSA (fPSA) level, reports of mpMRI examination, and results of prostate biopsy were extracted from clinical records. Prostate volume was measured using mpMRI examination, the ratio of fPSA (f/t PSA) was measured by dividing the (fPSA) by the tPSA, and the PSA density (PSAD) was calculated by dividing the tPSA by the prostate volume. All mpMRI examination were performed using the 3.0-T MRI system with a pelvic phased-array coil, complaint with European Society of Urology Radiology guidelines. The scan protocol for all patients included T2-weighted imaging, diffusion-weighted imaging, and dynamic contrast-enhanced imaging. The prostate mpMRI images were interpreted by two experienced genitourinary radiologists with at least three years of prostate mpMRI experience. The mpMRI results were divided into groups according to the reports: “negative”, “equivocal”, and “suspicious” for the presence of PCa (MRI-PCa), seminal vesicle invasion (MRI-SVI), lymph node invasion (MRI-LNI) according to the mpMRI reports.

All patients underwent transrectal ultrasound-guided systematic12-point biopsy according to the same protocol by three surgeons. If suspected malignant nodules by mpMRI and/or ultrasound, additional 1–5 needles were performed in regions with cognitive MRI-ultrasound fusion and/or abnormal ultrasound echoes. Biopsy cores were analyzed according to the standards of International Society of Urological Pathology.

### Machine learning-assisted methods

Four types of supervised machine learning-based methods (ANN, SVM, CART, and RF) were applied in this study. Nine variables comprising age, PSA derivates (tPSA, f/tPSA, and PSAD), prostate volume, mpMRI results (MRI-PCa, MRI-SVI, and MRI-LNI), and results of prostate biopsy were used to develop the PCa and CSPCa prediction models. Age of patients, PSA derivates, and prostate volume were normalized [(value − minimum value)/(maximum value − minimum value)] to fall in between 0 and 1, and entered as continuous variables. The mpMRI parameters were entered as dummy variables, and biopsy results were entered as binary variables.

The machine learning models were fit using the packages in R (version 3.6.2). The ANN is based on biological neural networks and composed of interconnected groups of artificial neurons [15]. And it was trained using the function of “Std_Backpropagation” in the package of “RSNNS”, and used three hyperparameters: size, learnFuncParams, and maxit. The three hyperparameters for ANN are c(3,3), 0.05 and 100 in the PCa model, and c(4,2), 0,05, and 100 in the CSPCa model. The SVM model is a machine learning model that finds an optimal boundary between the possible outputs. It was trained using the package of “e1071”, used a radical kernel and consisted of three hyperparameters: degree, cost, and gamma. The three hyperparameters for SVM are 3, 1, and 0.005 in the PCa model, and 3, 2 and 0.005 in the CSPCa model. The CART is based on the recursive partitioning method and belongs to a family of nonparametric regression methods. It was trained with the package of “rpart” and used three hyperparameters: minsplit, minbucket, and complexity parameter cp. The three hyperparameters for CART are 15, 5, and 0.01 in the PCa model, and 10, 3 and 0.01 in the CSPCa model. The RF is an ensemble learning method which that generating multiple decision trees and forming a “forest” to jointly determine output class [18]. The RF model was trained with using the package of “randomForest” in R, and used two hyperparameters: ntree, and mtry in this study. The two hyperparameters for RF are 500 and 2 in the PCa and CSPCa models.

### Statistical analysis

All data cleaning and analyses were conducted using R statistical software (Version 3.6.2). Diagnostic accuracy of the models was evaluated using the area under the ROC curve (AUC). The 95% confidence interval (CI) and comparisons of AUCs were determined using the method of DeLong et al. [19]. Performance characteristics of the models were examined by calibration plots. Calibration was assessed by grouping men in the validation cohort into delices (each of size 20 or 21), and then comparing the mean of predicated probabilities and the observed proportions. The sum squares of the residuals (SSR) was used to assess the deviation of calibration plots from the 45° line [20]. The clinical utility of the models was evaluated with a decision-curve analysis.

## Results

### Patient characteristics

A total of 688 cases were included in this study. The patients (480, 70%) biopsied before December 31, 2018 were used as training cohort, and the remaining patients (208, 30%) were used as validation cohort. Table 1 summarized the patient characteristics stratified by pathological results. PCa patients displayed higher age (70 vs 66 years, *P* < 0.001), tPSA (20.8 vs 10.5 ng/ml, *P* < 0.001), and PSAD (0.46 vs 0.18, *P* < 0.001), while lower f/tPSA (0.11 vs 0.15, *P* < 0.001) and prostate volume (38 vs 58 ml, *P* < 0.001) compared with no-PCa (Table 1). Additionally, the proportions for suspicious presence of PCa (73% vs 22%), SVI (31% vs 0.7%), and LNI (10% vs 0%) by mpMRI examination were higher among PCa patients than no-PCa (Table 1). The CSPCa patients displayed similar pattern with no-CSPCa patients (Table 1).

**Table 1:** The clinical characteristics of enrolled patients stratified by pathological results between April 2016 and March 2020

Clinical characteristics	PCa (GS ≥ 3 + 3)			CSPCa (GS ≥ 3 + 4)		
	No (n = 443)^†^	Yes (n = 245)^†^	*P*	No (n = 488)^†^	Yes (n = 200)^†^	*P*
Age (years)	66 (61–72)	70 (63–76)	< 0.001	66 (61–72)	70 (63–76)	< 0.001
tPSA (ng/ml)	10.5 (6.65–17.0)	20.8 (9.67–30.3)	< 0.001	10.9 (6.70–17.1)	23.0 (10.9–33.1)	< 0.001
f/tPSA	0.15 (0.10–0.21)	0.11 (0.07–0.17)	< 0.001	0.15 (0.10–0.21)	0.11 (0.07–0.17)	< 0.001
PSAD (ng/ml^2^)	0.18 (0.11–0.29)	0.46 (0.25–0.73)	< 0.001	0.19 (0.11–0.30)	0.52 (0.30–0.80)	< 0.001
PV (ml)	58 (39–82)	38 (27–58)	< 0.001	57 (38–81)	37 (27–54)	< 0.001
MRI-PCa, No. (%)			< 0.001			< 0.001
Negative	250 (56)	37 (15)		264 (54)	23 (12)	
Equivocal	95 (21)	29 (12)		104 (21)	20 (10)	
Suspicious	98 (22)	179 (73)		120 (25)	157 (79)	
MRI-SVI, No. (%)			< 0.001			< 0.001
Negative	439 (99)	163 (67)		481 (99)	121 (61)	
Equivocal	1 (0.2)	7 (3)		1 (0.2)	7 (4)	
Suspicious	3 (0.7)	75 (31)		6 (1)	72 (36)	
MRI-LNI, No. (%)			< 0.001			< 0.001
Negative	432 (98)	202 (82)		475 (97)	159 (80)	
Equivocal	11 (2)	18 (7)		12 (2)	17 (9)	
Suspicious	0 (0)	25 (10)		1 (0.2)	24 (12)	

*PCa* prostate cancer, *CSPCa* clinically significant prostate cancer, *GS* Gleason score, *tPSA* total prostate-specific antigen, *f/tPSA* free/total PSA, *PV* prostate volume, *SVI* seminal vesicle invasion, *LNI* lymph node invasion

^†^Data are presented as median (quartile range) unless other indicated

### Comparison of predictive accuracy between machine-learning models

**Fig. 1:**
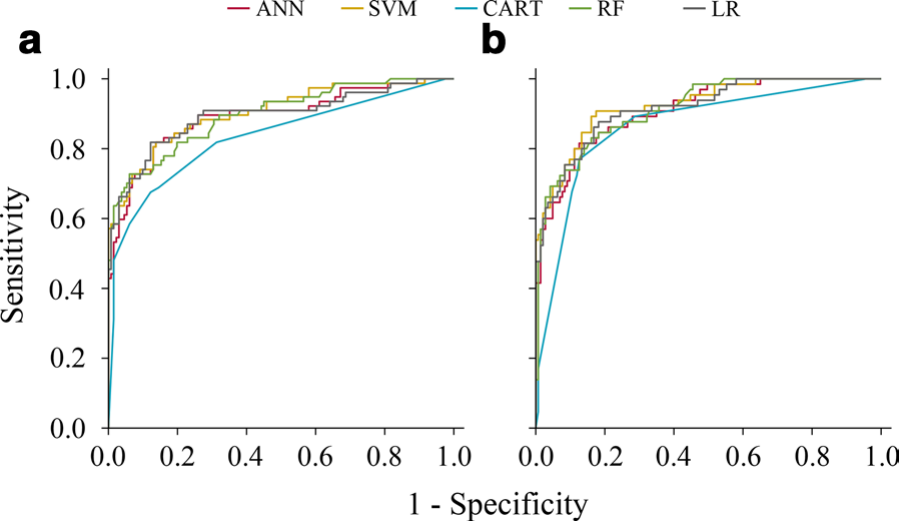
Receive operating characteristic (ROC) curves of machine-learning and logistic regression models for predicting prostate cancer (PCa) and clinically significant prostate cancer (CSPCa) in the validation cohort. **a** PCa: Gleason score ≥ 3 + 3; **b** CSPCa: Gleason score ≥ 3 + 4. *Abbreviations ANN* artificial neural network, *SVM* support vector machine, *CART* classification and regression tree, *RF* random forest, *LR* logistic regression

In our study, four machine-learning models based on age, PSA derivates, prostate volume, and mpMRI parameters were developed to predict initial biopsy results. Among these machine-learning assisted models for PCa and CSPCa, the SVM (AUC = 0.903 for PCa and AUC = 0.925 for CSPCa), RF (AUC = 0.897 for PCa and AUC = 0.916 for CSPCa), LR (AUC = 0.894 for PCa and AUC = 0.917 for CSPCa), and ANN (AUC = 0.891 for PCa and AUC = 0.911 for CSPCa) models outperformed CART (AUC = 0.834 for PCa and AUC = 0.867 for CSPCa) model in diagnostic accuracy (all *P* < 0.05); Whilst the pairwise comparison of AUCs were insignificant amongst ANN, SVM, RF, and LR models for PCa and CSPCa, respectively (each *P* > 0.05) (Fig. 1).

**Fig. 2:**
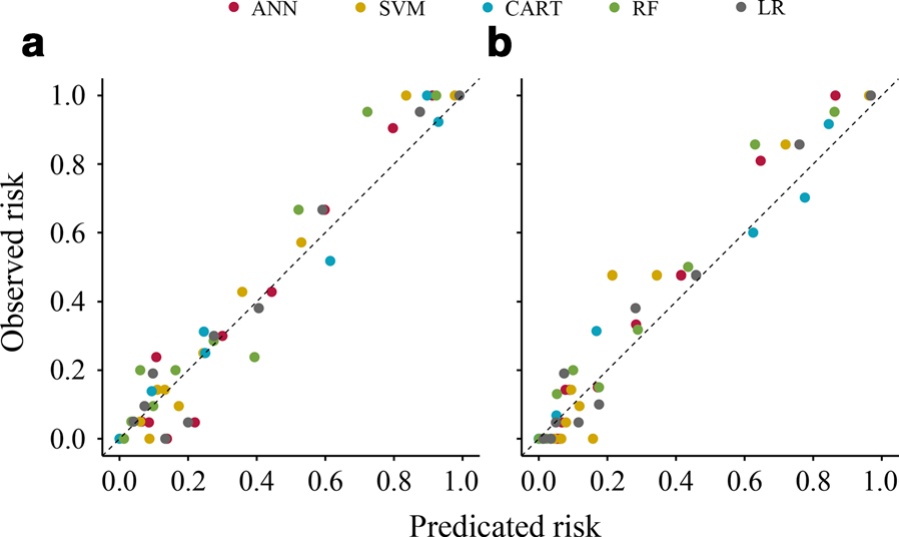
Calibration plot of observed vs predicted rick of prostate cancer (PCa) and clinically significant prostate cancer (CSPCa) using machine-learning and logistic regression models in the validation cohort. **a**: PCa: Gleason score ≥ 3 + 3; **b** CSPCa: Gleason score ≥ 3 + 4. *Abbreviations ANN* artificial neural network, *SVM* support vector machine, *CART* classification and regression tree, *RF* random forest, *LR* logistic regression

Regarding PCa models, the calibration plot of predicated probabilities against observed proportion of PCa indicated excellent concordance in CART model (SSR = 0.027), followed by SVM (SSR = 0.049), LR (SSR = 0.063), ANN (SSR = 0.091), and RF (SSR = 0.125) (Fig. 2a). For CSPCa models, the calibration plot of CART also had good agreement between the predicated probability and observed ratio of CSPCa on biopsy (SSR = 0.033), followed by LR (SSR = 0.046), ANN (SSR = 0.065), RF (SSR = 0.082), and SVM (SSR = 0.142) models (Fig. 2b).

### Impact of machine learning-assisted models on biopsies avoided

**Fig. 3:**
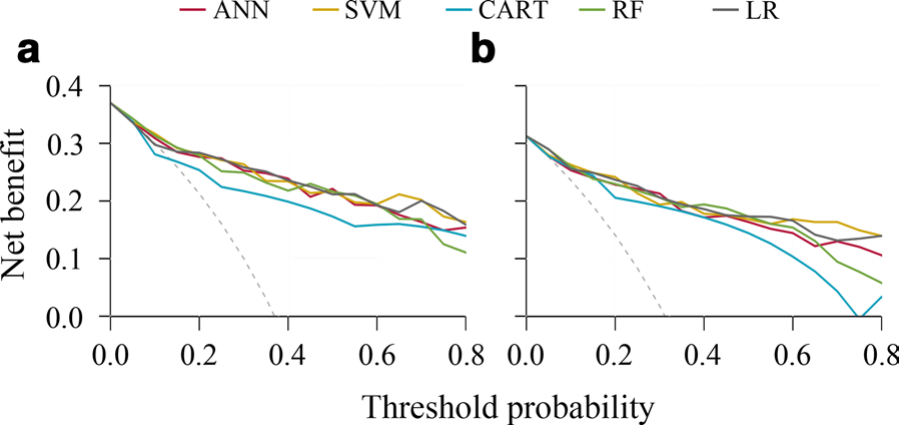
Decision curve analysis (DCA) of machine-learning and logistic regression models for predicting prostate cancer (PCa) and clinically significant prostate cancer (CSPCa) in the validation cohort. **a** PCa: Gleason score ≥ 3 + 3; **b** CSPCa: Gleason score ≥ 3 + 4. *Abbreviations ANN* artificial neural network, *SVM* support vector machine, *CART* classification and regression tree, *RF* random forest, *LR* logistic regression

To further assess potential clinical benefit of the machine learning-assisted models, we performed DCA using the predicated risk in the validation cohort. It was observed that the ANN, SVM, RF, and LR models for PCa had higher net benefit than CART model across the threshold probabilities above 5%, and the five models for CSPCa displayed similar net benefit across the threshold probabilities below 40% (Fig. 3).

Clinical consequences of using machine learning-assisted models at given sensitivity, including the number of biopsies that could be spared and the number of PCa by Gleason score that would be missed were displayed in Table 2. Using the SVM (74/143, 52%) and RF (76/143, 53%) models for PCa, significantly more unnecessary biopsies would be spared at 95% sensitivity for detection of CSPCa, compared with using ANN (53/143, 37%) and LR (50/143, 35%) models (all *P* < 0.05) (Table 2). Additionally, RF (81/143, 57%), SVM (79/143, 55%), and ANN (76/143, 53%) models for CSPCa spared more unnecessary biopsies than LR (67/143, 47%) model at 95% sensitivity (Table 2). At 95% sensitivity for detection of CSPCa, the RF and SVM models for CSPCa spared more unnecessary biopsies than the corresponding models for PCa (Table 2). However, the differences were insignificant (*P* = 0.688 for RF model, and *P* = 0.686 for SVM model).

**Table 2:** Percentage of biopsies that would be spared or delayed using machine-learning and logistic regression models at given sensitivity for detection of CSPCa in the validation cohorts

Models	Sensitivity for detection of CSPCa	Cut-off for predicted risk (%)	Biopsies sSpared ^†^ (n = 208), n (%)	Unnecessary biopsy avoided		Biopsy delayed		
				GS < 3 + 3 (n = 131), n (%)	GS = 3 + 3 (n = 12), n (%)	GS = 3 + 4 (n = 11), n (%)	GS = 4 + 3 (n = 18), n (%)	GS ≥ 4 + 4 (n = 36), n (%)
*Biopsies spared or delayed using PCa models at given sensitivity for detection of CSPCa*								
ANN	64/65 (98%)	9	34 (16)	32 (24)	1 (8)	0 (0)	1 (6)	0 (0)
SVM	64/65 (98%)	11	57 (24)	55 (42)	1 (8)	0 (0)	0 (0)	1 (3)
CART	64/65 (98%)	NA	NA	NA	NA	NA	NA	NA
RF	64/65 (98%)	7	61 (29)	57 (44)	3 (25)	1 (9)	0 (0)	0 (0)
LR	64/65 (98%)	7	30 (14)	27 (21)	2 (17)	1 (9)	0 (0)	0 (0)
ANN	62/65 (95%)	11	56 (25)	51 (39)	2 (17)	1 (9)	1 (6)	1 (3)
SVM	62/65 (95%)	14	77 (37)	71 (54)	3 (25)	1 (9)	1 (6)	1 (3)
CART	62/65 (95%)	NA	NA	NA	NA	NA	NA	NA
RF	62/65 (95%)	11	79 (38)	73 (56)	3 (25)	1 (9)	2 (11)	0 (0)
LR	62/65 (95%)	10	53 (25)	47 (36)	3 (25)	1 (9)	1 (6)	1 (3)
ANN	59/65 (91%)	27	109 (52)	99 (76)	4 (33)	3 (27)	1 (6)	2 (6)
SVM	59/65 (91%)	23	110 (53)	100 (76)	4 (33)	4 (36)	1 (6)	1 (3)
CART	57/65 (88%)	10	104 (50)	90 (69)	6 (50)	4 (36)	1 (6)	3 (8)
RF	59/65 (91%)	20	101 (49)	91 (69)	4 (33)	4 (36)	2 (11)	0 (0)
LR	59/65 (91%)	24	107 (51)	97 (74)	4 (33)	4 (36)	1 (6)	1 (3)
*Biopsies spared or delayed using CSPCa models at given sensitivity for detection of CSPCa*								
ANN	64/65 (98%)	7	60 (29)	58 (44)	1 (8)	0 (0)	0 (0)	1 (3)
SVM	64/65 (98%)	9	69 (33)	65 (50)	3 (25)	0 (0)	0 (0)	1 (3)
CART	64/65 (98%)	NA	NA	NA	NA	NA	NA	NA
RF	64/65 (98%)	7	79 (38)	75 (57)	3 (25)	1 (9)	0 (0)	0 (0)
LR	64/65 (98%)	6	61 (29)	57 (44)	3 (25)	0 (0)	1 (6)	0 (0)
ANN	62/65 (95%)	8	79 (38)	75 (57)	1 (8)	1 (9)	1 (6)	1 (3)
SVM	62/65 (95%)	10	82 (39)	76 (58)	3 (25)	1 (9)	1 (6)	1 (3)
CART	62/65 (95%)	NA	NA	NA	NA	NA	NA	NA
RF	62/65 (95%)	8	84 (40)	78 (60)	3 (25)	1 (9)	2 (11)	0 (0)
LR	62/65 (95%)	7	70 (34)	64 (49)	3 (25)	1 (9)	1 (6)	1 (3)
ANN	59/65 (91%)	9	96 (46)	86 (66)	4 (33)	4 (36)	1 (6)	1 (3)
SVM	59/65 (91%)	18	124 (60)	112 (85)	6 (50)	4 (36)	1 (6)	1 (3)
CART	58/65 (89%)	10	109 (52)	97 (74)	5 (42)	4 (36)	1 (6)	2 (6)
RF	59/65 (91%)	13	102 (49)	92 (70)	4 (33)	3 (27)	3 (17)	0 (0)
LR	59/65 (91%)	14	105 (50)	32 (24)	4 (33)	3 (27)	1 (6)	2 (6)

*PCa* prostate cancer, *CSPCa* clinically significant prostate cancer, *GS* Gleason score, *ANN* artificial neural network, *SVM* support vector machine, *CART* classification and regression tree, *RF* random forest, *LR* logistic regression, *NA* not applicable

^†^Number of biopsies spared = number of unnecessary biopsy avoided + number of biopsy delayed

## Discussion

In our study, we developed, validated, and compared the machine learning-assisted models with LR analysis to predict PCa and CSPCa among patients with serum tPSA ≤ 50 ng/ml, using the same variables and population. The ANN, SVM, and RF models yielded similar diagnostic accuracy and net benefit with LR, and CART had lower diagnostic accuracy than LR in prediction of PCa and CSPCa. However, the CART model illustrated best calibration for PCa and CSPCa. And the SVM and RF models for PCa and CSPCa spared more biopsies than LR at 95% sensitivity for detection of CSPCa.

PCa was detected in 20% of the subjects with serum tPSA in the gray zone (4–10 ng/ml) in our study (data not shown). This was similar with the PCa detection rates of the same group of patients in Singapore (21%) [21], Japan (20%) [22], and Korea (20%) [23], while lower than that in Cleveland Clinic (40%) and Durham VA hospital (43%) [24]. This may suggest that the relationship between PCa risk and PSA level varies between Asian and Western populations, and it is essential to establish area-based risk prediction models. Our study revealed that the rates of PCa and CSPCa increased with tPSA, and CSPCa were detected in 279/301 (93%) of the men with serum tPSA > 50 ng/ml. Therefore, we recommended all cases with tPSA > 50 ng/ml to undergo prostate biopsy, and developed machine learning-assisted models to predict PCa and CSPCa among patients with tPSA ≤ 50 ng/ml (in accordance with ERSPC-RC) [10].

A growing body of literatures have validated the clinical utility of mpMRI in the detection and localization of CSPCa [4]. However, as far as we know, the knowledge about the performance of risk prediction models incorporating mpMRI parameters is limited. We developed machine learning-assisted models based on age, PSA derivates, prostate volume, and mpMRI parameters in our study. The digital rectal examination and transrectal ultrasound were excluded as risk factors because of potential interobserver variability in its assessment [3, 25]. The ANN, SVM, RF and LR models (AUC = 0.891–0.903 for PCa, and AUC = 0.911–0.925 for CSPCa) incorporating mpMRI parameters developed in our study outperformed CRCC-PC (AUC = 0.80 for PCa, and AUC = 0.83 for CSPCa) and MRI-ERSPC-RC (AUC = 0.85 for CSPCa). This may suggest that the combination of mpMRI parameters including MRI-PCa, MRI-SVI, and MRI-LNI could improve the diagnostic accuracy of prediction model for PCa and CSPCa. The mpMRI parameters included in our models were extracted from the reports of mpMRI examination and were somewhat subjective. Some study showed that mpMRI radiomics features significantly associated with PCa aggressiveness on the histopathological and genomic levels [26, 27]. And addition of mpMRI radiomics may enhance the objectivity and diagnostic accuracy of prediction model.

For prediction of PCa, ANN has become (alongside LR) one of the fastest growing and most effective machine-learning algorithms [15]. Theoretically, ANN has considerable advantages over traditional statistical approaches, which automatically allow no explicit distributional assumptions, arbitrary nonlinear associations, and possible interactions. A systematic review including 28 studies showed that ANN outperformed regression in 10 (36%) cases, ANN and regression tied in 14 (50%) cases, and regression wined in the remaining 4 (14%) cases [14]. In our study, ANN displayed similar diagnostic accuracy and net benefit for prediction of PCa and CSPCa with LR. Based on the available data, ANN does not have significantly advantages in clinical practice compared with LR, and should not replace traditional LR for the classification of medical data.

Another three machine-learning algorithms (SVM, CART, and RF) were developed to predict PCa and CSPCa in our study. Some studies showed that RF algorithms outperformed LR model in the fields of identifying peripheral artery disease and mortality risk [28], predicting clinical outcomes after robot-assisted radical prostatectomy [29], and predicting clinical outcomes of large vessel occlusion before mechanical thrombectomy [30]. The RF and SVM showed similar diagnostic accuracy with LR model in prediction of PCa and CSPCa in our study, while spared more unnecessary biopsies than LR model at given sensitivity of 98% or 95% (Table 2). Above all, our study did not have enough power to draw conclusion that ANN, SVM, CART and RF models outperformed traditional LR analysis in diagnostic of CSPCa. Now we are entering the era of big data, in which complete patient data including macro-level physiology and behavior, laboratory and imaging studies, and “-omic” data, are becoming more readily available. Machine learning may become an indispensable tool to handle the complex data [6]. Further validation is required.

## Conclusions

Our study developed and compared machine-learning models with LR analysis to predict PCa and CSPCa. The SVM and RF models yielded similar diagnostic accuracy and net benefit with LR, while spared more unnecessary prostate biopsies than LR model at 95% sensitivity for detection of CSPCa. CART model illustrated best calibration for the prediction of PCa and CSPCa. Our study did not have sufficient power to draw conclusion that machine-learning models outperformed traditional LR analysis in prediction of PCa and CSPCa. All methods should continue to be used and explored in complementary ways.

## Data Availability

The datasets used and/or analyzed during the current study are available from the corresponding author on reasonable request.

## Abbreviations

PCa: Prostate cancer
CSPCa: Clinically significant prostate cancer
PSA: Prostate-specific antigen
tPSA: Total prostate-specific antigen
LR: Logistic regression
ANN: Artificial neural network
SVM: Support vector machine
CART: Classification and regression tree
RF: Random forest
mpMRI: Multiparametric magnetic resonance imaging
f/t PSA: Free/total prostate-specific antigen
SVI: Seminal vesicle invasion
LNI: Lymph node invasion
DCA: Decision curve analysis
AUC: Area under the curve
CI: Confidence interval
SSR: Sum squares of the residuals
